# Comparison of rehabilitation outcomes between robot-assisted and freehand screw placement in treatment of femoral neck fractures: a systematic review and meta-analysis

**DOI:** 10.1186/s12891-024-07325-0

**Published:** 2024-03-08

**Authors:** Yiyang Li, Yan Wang, Benchao Dong, Peichuan Yang, Yadi Sun, Liyun Zhou, Jiahui Shen, Xinlong Ma, Jianxiong Ma

**Affiliations:** 1 Tianjin Hospital of Tianjin University, Tianjin, 300211 People’s Republic of China; 2 Tianjin Orthopedic Institute, Tianjin, 300211 People’s Republic of China; 3 Tianjin Key Laboratory of Orthopedic Biomechanics and Medical Engineering, Tianjin, 300211 People’s Republic of China

**Keywords:** Femoral neck fractures, Rehabilitation, Robotic surgery, Systematic review

## Abstract

**Purpose:**

To compare the postoperative rehabilitation of femoral neck fractures treated with robot-assisted nailing and freehand nailing.

**Methods:**

We systematically searched the PubMed, EMBASE, Cochrane, China National Knowledge Infrastructure(CNKI), WanFang database, China Science and Technology Journal Database (VIP) and Web of Science databases to identify potentially eligible articles. Indispensable data such as the year of publication, country, study type, robot type, age, number of patients, sex distribution, study design, and outcome indicators were extracted. The outcome indicators of interest included healing rate, length of healing time, Harris score, operation time, frequency of X-ray fluoroscopy, frequency of guide pin insertion, and intraoperative blood loss. RevMan 5.4.1 was used for the meta-analysis.

**Results:**

Fourteen studies with 908 participants were included in this meta-analysis. The results showed that in terms of healing rate (SMD = 2.75, 95% CI, 1.03 to 7.32, *P* = 0.04) and Harris score (SMD = 2.27, 95% CI, 0.79 to 3.75, *P* = 0.003), robot-assisted screw placement technique scores were higher than the traditional freehand technique. Additionally, operative time (SMD = -12.72, 95% CI, -19.74 to -5.70, *P* = 0.0004), healing time (SMD = -13.63, 95% CI, -20.18 to -7.08, *P* < 0.0001), frequency of X-ray fluoroscopy (SMD = − 13.64, 95% CI, − 18.32 to − 8.95, *P* < 0.00001), frequency of guide pin insertion (SMD = − 7.95, 95% CI, − 10.13 to − 5.76, *P* < 0.00001), and intraoperative blood loss (SMD = − 17.33, 95% CI, − 23.66 to − 11.00, *P* < 0.00001) were lower for patients who underwent robotic-assisted screw placement than those for patients who underwent the conventional freehand technique.

**Conclusion:**

Compared to the freehand nailing technique, robot-assisted nailing helps improve postoperative healing rates in patients with femoral neck fractures; shortens healing times; better restores hip function; reduces the number of intraoperative fluoroscopies, guides pin placements; reduces intraoperative bleeding; and increases perioperative safety.

**Supplementary Information:**

The online version contains supplementary material available at 10.1186/s12891-024-07325-0.

## Introduction

Femoral neck fractures are the most common hip fractures, accounting for 3–4% of all fractures and 50–60% of hip fractures [1, 2]. Currently, the mainstream treatment modalities include conservative treatment, internal fixation, external fixation, and hip arthroplasty. In clinical practice, there is a general consensus for early surgical treatment of patients with femoral neck fractures, as conservative treatment requires patients to be bedridden for long periods and carries the risk of infection and thrombosis [3]. Closed reduction with percutaneous hollow tension screw internal fixation is one of the main procedures used to treat femoral neck fractures [4, 5]. This method uses closed reduction, which avoids excessive medically induced injuries, and its three inverted triangular arrangements of screws [6] allow for dynamic compression, ensuring secure fixation while ensuring effective fixation [7]. However, this procedure relies on the surgeon’s experience in placing screws using X-ray fluoroscopy, which is not sufficiently precise, requires a high level of skill, and exposes the surgeon to high radiation doses. From a patient-healing perspective, the precision of freehand nail placement can also affect femoral neck healing. Schep et al. [8] found that the precise position and orientation of the intraoperative fixation screw are closely related to the prognosis of fracture stability, the occurrence of re-displacement, and fracture healing time. In contrast, the traditional C-arm-assisted freehand nail placement method not only requires the surgeon to constantly adjust the position of the C-arm, but also increases the risk of contamination of the operative area. Moreover, because of the limited precision of freehand nail placement, repeated drilling and puncturing can affect blood supply and bone destruction in cases of nail placement failure [9, 10], which in turn affects the healing rate [11]. Despite extensive efforts to improve and investigate the use of internal fixation for femoral neck fractures, an optimal fixation method has yet to be identified, and the incidence of postoperative osteonecrosis and femoral head necrosis remains high [12]. Accurate and minimally invasive screw placement while reducing radiation exposure to patients and surgeons during surgery, has become an urgent challenge. Currently, with the popularity of 2D and 3D digital imaging, computer-guided and robotic surgical systems assisted by minimally invasive internal fixation are increasingly used for the treatment of femoral neck fractures. Compared to traditional surgery, robot-assisted surgery has the advantages of shorter surgical time and less radiation damage; therefore,, so it is favoured by an increasing number of orthopaedic surgeons and is gradually being used in clinical practices [13, 14]. Orthopaedic surgical robots provide data analysis and processing, surgical navigation, simulation planning, and precise positioning, and can place screws quickly, accurately, and safely; several studies have concluded that the accuracy of nail placement is over 98% [15]. Although robot-assisted nail placement has been shown to be superior to conventional nail placement in terms of accuracy, there is a lack of evidence that robot-assisted nail placement results in a better prognosis for patients with femoral neck fractures. Exploring the close relationship between robot-assisted surgery and patient prognosis is currently the focus of clinical research in robot-assisted femoral neck fracture surgeries [16]. Previous clinical studies have generally concluded that robot-assisted surgery has significant advantages [17, 18] in terms of short operative time, reduced number of fluoroscopic views and guide pin placements, and reduced intraoperative bleeding, and that these advantages facilitate the postoperative rehabilitation of patients. However, in terms of healing rate, healing time, and postoperative Harris score, several studies concluded that there was no difference between the robot-assisted nail placement technique and the traditional freehand nail placement technique, while some studies concluded that patients treated with robot-assisted surgery had a better prognosis. In conclusion, there is controversy among researchers regarding whether robot-assisted nailing provides better rehabilitation outcomes in patients with femoral neck fractures. This study aimed to summarise previous studies through a meta-analysis to verify the advantages of robot-assisted nail placement in intraoperative operations and to determine whether robot-assisted nail placement improves the rehabilitation outcomes of patients with femoral neck fractures. The indicators used to evaluate rehabilitation outcomes were healing rate, healing time, and postoperative Harris score.

## Methods

### Data search strategy

This meta-analysis followed the Preferred Reporting Items for Systematic Reviews and Meta-Analysis (PRISMA) procedure [19]. We systematically searched PubMed, EMBASE, Cochrane, China National Knowledge Infrastructure (CNKI), WanFang database, China Science and Technology Journal Database (VIP), and Web of Science to identify potentially eligible articles. Notably, these databases were updated on January 19, 2024. We used the following keywords: ‘‘Robotics’’, “Robot”, “Robot-assisted”, ‘‘Fracture’’, “Femoral Neck Fractures”, and ‘‘Fracture Healing’’. For example, the search strategy employed for PubMed is presented in Table 1. Two reviewers (Li Yiyang and Sun Yadi) independently searched all the titles and abstracts, and the references of relevant studies were reviewed for additional valuable literature. Any divergence was resolved through discussion or consultation with a third reviewer (Wang Yan).

**Table 1:** Search strategy for Pubmed

**Database**	**Search strategy**
**Pubmed**	(“Fractures,Bone“[Mesh] OR “Fracture Healing“[Mesh] OR “Fracture Fixation, Intramedullary“[Mesh] OR “Fracture Fixation, Internal“[Mesh] OR “Fracture Fixation“[Mesh] OR “Open Fracture Reduction“[Mesh] OR “Closed Fracture Reduction“[Mesh]) AND (“Robotics“[MeSH Terms] OR “robot“[All Fields] OR “robotics“[All Fields] OR” robotic“[All Fields])

We used the Population, Intervention, Comparison, Outcomes and Study (PICOS) system [20] for this systematic review. This system frames the review’s aim, search strategy, and study inclusion and exclusion criteria. The critical items of our systematic review are described below:

- P (Population): Patients with femoral neck fractures.
- I (Intervention): Robot-assisted screw placement.
- C (Comparison): Freehand screw placement.
- O (Outcome): Healing rate, length of healing time, Harris score, operation time, frequency of X-ray fluoroscopy, frequency of guide pin insertion, and intraoperative blood loss.
- S (Study design): Randomised controlled trial and cohort studies.

### Study selection

Inclusion criteria were identified before the search, and the following criteria were used: articles involving robot-assisted femoral neck screw placement, quantitative indicators for evaluating patient rehabilitation outcomes, and providing sufficient data for meaningful comparison (> 10 patients per study group). The exclusion criteria were duplicate publications and articles without traditional freehand screw placement in the control group. Furthermore, only human studies were considered. The inclusion of studies was not limited by study size or publication type, and the excluded publications were review articles and commentaries.

### Quality assessment and data extraction

Two reviewers independently assessed all included studies using the risk-of-bias tool. Retrospective cohort studies were evaluated using the Newcastle-Ottawa Quality Assessment Scale, which is rated using 0–9 stars. Seven or more stars indicate sufficiently high quality. The Cochrane risk-of-bias criteria were used to assess the quality of the randomized clinical trial (RCT) regarding selection, performance, detection, attrition, reporting, and other biases. We defined other biases as the difference in baseline characteristics between the experimental and control groups.

Two reviewers independently extracted the data extraction. Any disagreements were resolved through discussion or consultation with a third reviewer. Indispensable data, such as publication year, study type, age, number of patients, gender distribution, study design, and outcomes, were extracted. Outcome indicators of interest included healing rate, length of healing time, Harris score, operation time, frequency of X-ray fluoroscopy, frequency of guide pin insertion and intraoperative blood loss.

## Results

### Characteristics of the included studies

**Fig. 1:**
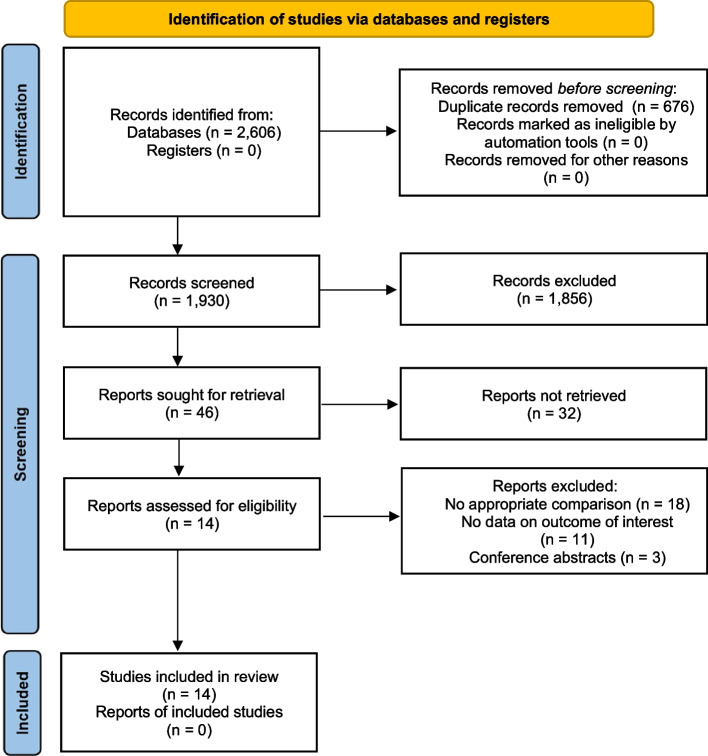
Schematic diagram of the study procedure

**Fig. 2:**
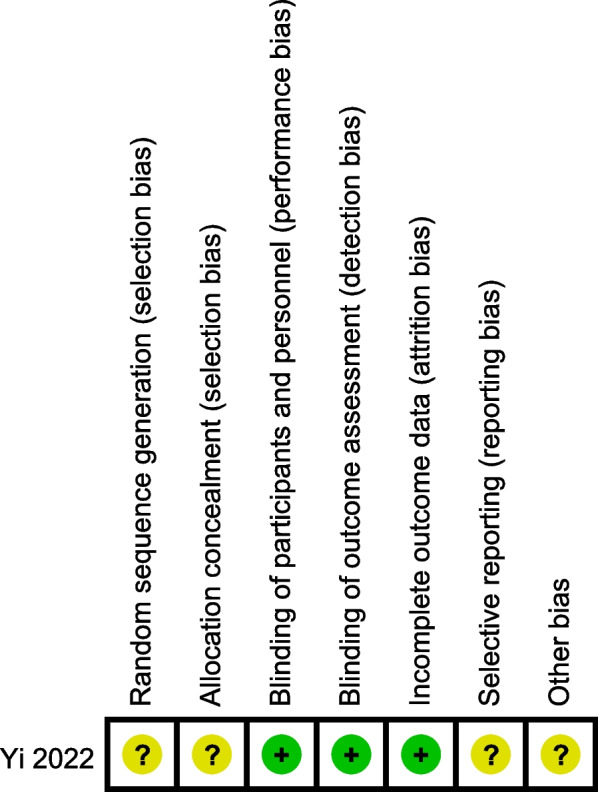
The risk of bias of the included randomised controlled trial

Figure 1 shows the process of study inclusion. Notably, 2606 relevant studies were obtained through a web search. In total, 676 studies were excluded because they were duplicates. After assessing the titles and abstracts, 1930 studies were excluded because their content did not meet the criteria. After verifying the full text of the remaining 46 studies, 13 retrospective cohort studies (RCS) [6, 17, 18, 21–30] and one RCT [30] with 908 patients were finally included in this meta-analysis. The RCT was shown to have a low risk of bias in Fig. 2. Table 2 summarises the main characteristics of the included studies. Baseline information was balanced and comparable across the 14 studies. The cohort studies all had evaluation scores greater than seven stars (Table 3), and all the included studies demonstrated satisfactory quality.

**Table 2:** Characteristics of included studies

Study	Country	Study design	Mean follow-up period (month)	Mean age, y		No.of patients	Sex (M/F)		Outcome indicators
	Robot type			RA	FH		RA	FH	
Cao 2017 [23]	China	RCS	14.7	44.7	47.9	*N* = 56 RA = 20 FH = 36	10/10	19/17	Healing rate; Length of healing time; Harris score; Operation time; Frequency of X-ray fluoroscopy; Frequency of guide pin insertion; Intraoperative blood loss
	Universal Robots								
Chen 2023 [28]	China	RCS	31.4	43.6 ± 13.7	45.7 ± 12.7	*N* = 68 RA = 32 FH = 36	18/14	17/19	Harris score; Operation time; Frequency of X-ray fluoroscopy; Frequency of guide pin insertion; Intraoperative blood loss
	TiRobot								
Duan 2019 [26]	China	RCS	13.6	61.7 ± 5.2	62.1 ± 4.1	*N* = 49 RA = 26 FH = 23	11/15	9/14	Healing rate; Length of healing time; Harris score; Operation time; Frequency of X-ray fluoroscopy; Frequency of guide pin insertion; Intraoperative blood loss
	TiRobot								
Huang 2017 [18]	China	RCS	19.6	59.4 ± 5.6	59.1 ± 4.9	*N* = 64 RA = 32 FH = 32	10/22	12/20	Length of healing time; Harris score; Operation time; Frequency of X-ray fluoroscopy; Frequency of guide pin insertion; Intraoperative blood loss
	Bi⁃planar robot								
Huang 2023 [29]	China	RCS	22.2	48.2 ± 11.9	48.5 ± 9.8	*N* = 53 RA = 25 FH = 28	11/14	12/16	Length of healing time; Harris score; Operation time; Frequency of X-ray fluoroscopy; Frequency of guide pin insertion; Intraoperative blood loss
	TiRobot Advance								
Jing 2022 [22]	China	RCS	7.0	55.2	55	*N* = 74 RA = 31 FH = 43	11/20	14/29	Healing rate; Length of healing time; Harris score; Intraoperative blood loss
	TiRobot								
Lei 2021 [25]	China	RCS	6.0	51.86 ± 4.89	51.33 ± 4.3	*N* = 42 RA = 21 FH = 21	12/9	14/7	Healing rate; Length of healing time; Harris score; Operation time; Frequency of X-ray fluoroscopy; Frequency of guide pin insertion; Intraoperative blood loss
	TiRobot								
Liao 2022 [30]	China	RCS	8.0	44.1 ± 8.7	48.8 ± 8.0	*N* = 28 RA = 14 FH = 14	6/8	7/7	Length of healing time; Harris score; Operation time; Frequency of X-ray fluoroscopy
	TiRobot II								
Liu 2015 [24]	China	RCS	12.5	65.2 ± 4.2	60.5 ± 5.1	*N* = 46 RA = 21 FH = 25	8/13	11/14	Length of healing time; Harris score; Operation time; Frequency of X-ray fluoroscopy; Frequency of guide pin insertion; Intraoperative blood loss
	GD-2000								
Nie 2023 [17]	China	RCS	14.9	56.00 ± 4.22	54.87 ± 4.81	*N* = 41 RA = 18 FH = 23	8/10	10/13	Harris score; Operation time; Frequency of X-ray fluoroscopy; Frequency of guide pin insertion; Intraoperative blood loss
	TiRobot								
Tong 2016 [21]	China	RCS	18.0	47.5	51.5	*N* = 38 RA = 20 FH = 18	12/8	11/7	Healing rate; Length of healing time; Harris score; Operation time; Frequency of X-ray fluoroscopy; Frequency of guide pin insertion; Intraoperative blood loss
	TiRobot								
Wang 2019 [6]	China	RCS	12.0	49.03 ± 8.23	49.80 ± 7.68	*N* = 128 RA = 63 FH = 65	30/33	31/34	Healing rate; Harris score; Operation time; Frequency of X-ray fluoroscopy; Frequency of guide pin insertion; Intraoperative blood loss
	TiRobot								
Yi 2022 [31]	China	RCT	18.0	58.5 ± 6.3	57.5 ± 5.3	*N* = 68 RA = 32 FH = 36	19/13	16/20	Length of healing time; Harris score; Operation time
	TINAVI								
Zhu 2021 [27]	China	RCS	38.8	47.9 ± 13.5	47.7 ± 12.6	*N* = 153 RA = 50 FH = 83	26/24	47/36	Healing rate; Harris score; Operation time; Frequency of X-ray fluoroscopy; Intraoperative blood loss
	TiRobot

**Table 3:** Risk of bias assessment of the cohort studies

**RCS**	**Selection**	**Comparability**	**Outcome**	**Total**
**Cao 2017** [23]	✩ ✩ ✩	✩	✩✩✩	✩✩✩✩✩✩✩
**Chen 2023** [28]	✩ ✩ ✩ ✩	✩	✩✩✩	✩✩✩✩✩✩✩✩
**Duan 2019** [26]	✩ ✩ ✩ ✩	✩	✩✩✩	✩✩✩✩✩✩✩✩
**Huang 2017 **[18]	✩ ✩ ✩	✩	✩✩✩	✩✩✩✩✩✩✩
**Huang 2023** [29]	✩ ✩ ✩ ✩	✩	✩✩✩	✩✩✩✩✩✩✩✩
**Jing 2022** [22]	✩ ✩ ✩ ✩	✩	✩✩	✩✩✩✩✩✩✩
**Lei 2021 **[25]	✩ ✩ ✩	✩	✩✩✩	✩✩✩✩✩✩✩
**Liao 2022** [30]	✩ ✩ ✩✩	✩	✩✩✩	✩✩✩✩✩✩✩✩
**Liu 2015** [24]	✩ ✩ ✩	✩	✩✩	✩✩✩✩✩✩✩
**Nie 2023** [17]	✩ ✩ ✩	✩	✩✩✩	✩✩✩✩✩✩✩
**Tong 2016** [21]	✩ ✩ ✩	✩	✩✩✩	✩✩✩✩✩✩✩
**Wang 2019** [6]	✩ ✩ ✩	✩	✩✩✩	✩✩✩✩✩✩✩
**Zhu 2021** [27]	✩ ✩ ✩ ✩		✩✩✩	✩✩✩✩✩✩✩

### Healing rate and length of healing time

**Fig. 3:**
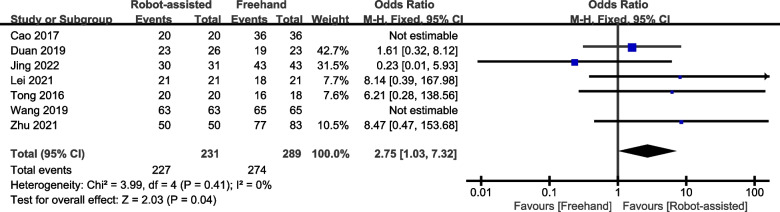
Forest plot of robot-assisted techniques versus conventional freehand techniques in femoral neck screws placement for healing rate

**Fig. 4:**
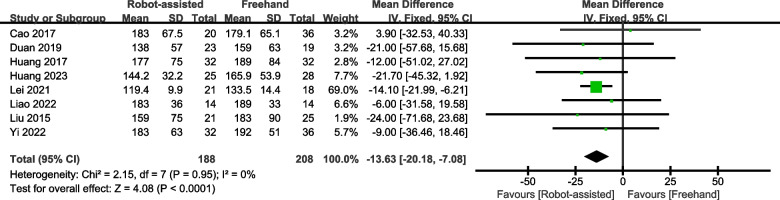
Forest plot of robot-assisted techniques versus conventional freehand techniques in femoral neck screws placement for the length of healing time

Seven studies provided data on the healing rate, and five studies provided data on the healing time. The results show that a higher percentage of patients recovered from robotic-assisted surgery (standardised mean difference (SMD) = 2.75, 95% confidence interval (CI), 1.03–7.32, *P* = 0.04; Fig. 3) with a shorter healing time (SMD = -13.63, 95% CI, -20.18 – -7.08, *P* < 0.0001; Fig. 4) than from unassisted surgery.

### Harris score

**Fig. 5:**
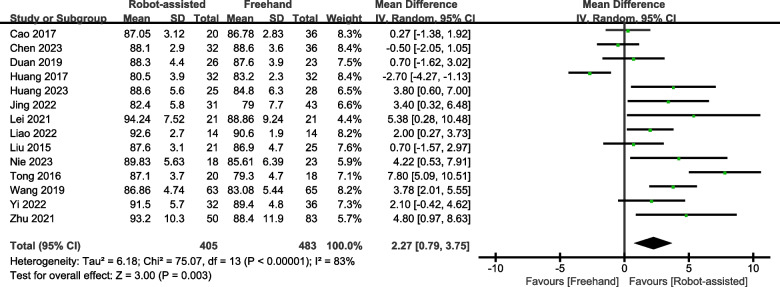
Forest plot of robot-assisted techniques versus conventional freehand techniques in femoral neck screws placement for Harris score

Ten studies provided data on the Harris score. The results showed that the Harris score for patients who underwent robotic-assisted screw placement (SMD = 2.27, 95% CI, 0.79–3.75, *P* < 0.003; Fig. 5) was greater than that of those who underwent the conventional freehand technique.

#### Operation time

**Fig. 6:**
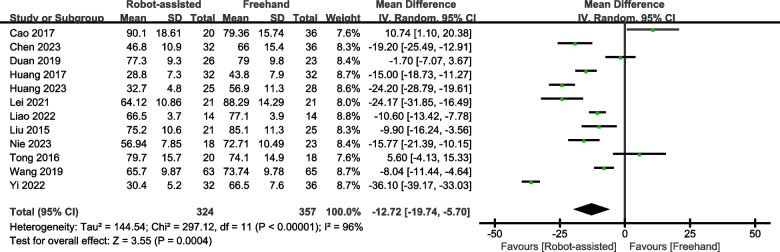
Forest plot of robot-assisted techniques versus conventional freehand techniques in femoral neck screws placement for operation time

Eight studies provided data on operation time. The results showed that the robot-assisted technique requires less operation time (SMD = -12.72, 95% CI, -19.74 – -5.70, *P* < 0.0004; Fig. 6) than the freehand technique.

### Frequency of X-ray fluoroscopy

**Fig. 7:**
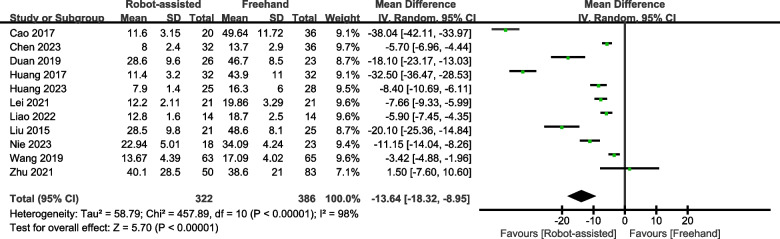
Forest plot of robot-assisted techniques versus conventional freehand techniques in femoral neck screws placement for frequency of X-ray fluoroscopy

Eight studies provided data on the frequency of X-ray fluoroscopy. The results showed that patients undergoing robot-assisted surgery require less fluoroscopy (SMD = -13.64, 95% CI, -18.32 – -8.95, *P* < 0.00001; Fig. 7) than those undergoing traditional surgery.

### Frequency of guide pin insertion

**Fig. 8:**
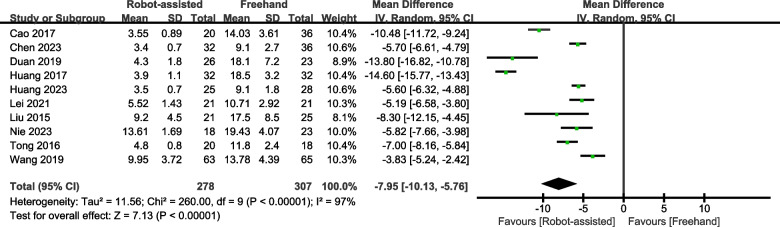
Forest plot of robot-assisted techniques versus conventional freehand techniques in femoral neck screws placement for frequency of guide pin insertion

Eight studies provided data on the frequency of guide pin insertion. The results showed that the robot-assisted technique requires fewer guide pin insertions (SMD = -7.95, 95% CI, -10.13 – -5.76, *P* < 0.00001; Fig. 8) than the freehand technique.

### Intraoperative blood loss

**Fig. 9:**
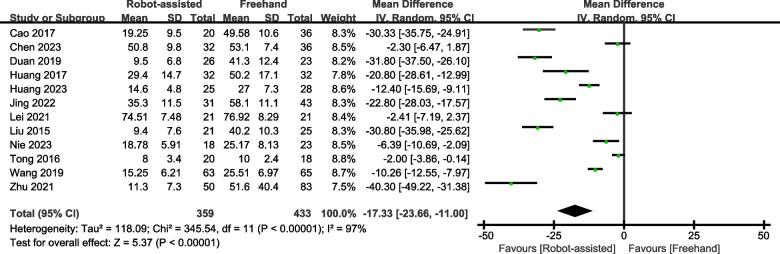
Forest plot of robot-assisted techniques versus conventional freehand techniques in femoral neck screws placement for intraoperative blood loss

Ten studies provided data on intraoperative blood loss. The results showed that patients with femoral neck fractures treated with robot-assisted surgery have less intraoperative blood loss (SMD = -17.33, 95% CI, -23.66 – -11.00, *P* < 0.00001; Fig. 9).

### Sensitivity analysis and publication bias test

In case of significant heterogeneity in the Harris score, operation time, frequency of X-ray fluoroscopy, frequency of guide pin insertion, and intraoperative blood loss results, a sensitivity analysis was conducted by omitting one study in each turn, and then the others were analysed to estimate whether a single study markedly affected heterogeneity. This analysis confirmed the stability of the Harris score, operation time, frequency of X-ray fluoroscopy, frequency of guide pin insertion, and intraoperative blood loss results.

Funnel plots were performed to test for possible publication bias. Supplemental material 1 shows that the studies were nearly symmetrically distributed on both sides of the vertical line, indicating a relatively small publication bias.

## Discussion

This meta-analysis showed that robot-assisted screw placement significantly improved patient recovery and reduced intraoperative injuries compared with traditional freehand screw placement. Femoral neck fractures are currently increasing in middle-aged and older adults, and ischemic necrosis of the femoral head and non-union of the fracture remain major postoperative complications [32]. Weil et al. [33] reported that in surgeries for femoral neck fractures, selecting an appropriate implantation site and depth is crucial. Wang et al. [6] also showed that the direction and position of intraoperative hollow screw placement are closely associated with the re-displacement, fixed stability, and healing of the fractures postoperatively. The more accurate the hollow screw placement, the more stable the internal fixation of the femoral neck fracture, and the lower the risk of fracture bone discontinuity [34]. Robots are increasingly used in femoral neck repair surgery because of their accuracy. Experimental evidence shows that robot-assisted femoral neck repair surgery is more accurate than freehand nail placement. Zhu et al. [27] and He et al. [35] mention that the screw parallelism in the robot-operated group was better than that in the freehand-operated group. However, the prognostic outcomes of the robot- and freehand-operated groups have been evaluated differently in different studies, with Zhu et al. [27] concluding that patients in the robot-operated group had a higher postoperative healing rate, and Wan et al. [25] concluding that both groups showed no statistical difference regarding healing rates. This is inconsistent with previous studies theory that states that “greater precision in nail placement leads to a better prognosis.” Therefore, a meta-analysis was necessary to pool past studies.

Our research showed that robot-assisted treatment of femoral neck fractures leads to a better prognosis. The healing rate and time may be associated with the precise nail placement of the robot. Robotic precision nail placement leads to higher parallelism and discrete rates [35]. Zhou et al. [36] demonstrated that a standard, inverted triangle distribution and good parallelism can promote fracture healing. Regarding the operative length, the analysis showed that robotic surgery was significantly shorter than conventional surgery. However, other studies arrived at a contradictory conclusion. Cao et al. [23] suggested that the robot requires more time. Nassim et al. [37] and Zheng et al. [38] concluded that the robotic and freehand screw placement showed no difference in operative time. These studies indicated that there is still room for improvement in reducing the operative time with robot-assisted technology. Arand et al. [39] also cited experience as vital in the length of surgery, as the actual intraoperative needle may shift to a certain extent when it encounters resistance, and less experienced operators may spend more time adjusting the needle position. Notably, other studies have attributed the prolonged operative time to the operative steps of the robot-assisted procedure; however, Duan et al. [26] suggest that most of the time spent in robot-assisted surgery is spent on device placement and commissioning, image acquisition, and other non-invasive procedures. Furthermore, Cao et al. [23] suggest in more detail why robot-assisted techniques take longer in operative steps. These include longer image acquisition time, sometimes requiring multiple x-rays; longer image transfer time, with images currently acquired by the C-arm needing to be copied to the system workstation for path planning; and the fact that most systems do not support simultaneous planning of multiple screw paths, with only one screw being placed in a single pass.

Notably, most studies agree that robotic-assisted surgery is less invasive than conventional surgery in the number of X-ray fluoroscopies, intraoperative bleeding, and guide needle placements, providing strong evidence that robotic-assisted surgery helps patient recovery. Robotic-assisted techniques do not require repeated fluoroscopy to determine needle placement [21] and can significantly reduce the number of fluoroscopies. Semi-automated needle placement results in fewer errors and significantly fewer needle placements than unassisted placement, facilitating the healing of the patient’s incision. In middle-aged and elderly patients with reduced bone mass, repeated pin placement can cause punctate loss of bone in the access area, preventing the pin from being placed in the right place and affecting the accuracy of internal fixation placement. Such subtle changes may not be accurately analysed from radiographic data but can affect long-term outcomes [22]. Similarly, fewer needle placements result in less bleeding, which benefits the early postoperative recovery of older patients, especially those with poor systemic organ function, and improves perioperative safety.

The application of orthopaedic robots in traumatic orthopaedics is still new compared with spinal and joint replacement surgeries, and robot-assisted femoral neck fracture treatment is currently the scenario that most commonly uses robots in traumatic orthopaedics. Therefore, studying the clinical outcomes and rehabilitation results of patients undergoing this kind of surgery is significant in selecting treatment modalities for femoral neck fractures and has reference values for developing orthopaedic robots. We believe that applying robotics to femoral neck fractures will ultimately improve the healing rate and shorten the healing time. This study determined the statistical differences between robot-assisted femoral neck fracture surgery and the freehand nail placement technique regarding healing rate, healing time, and postoperative Harris scores. The results showed that the robot-assisted technique was more effective than the unassisted screw placement technique in improving healing rates, reducing healing times, and improving patients’ postoperative Harris scores. This study also demonstrated the advantages of robotics in reducing the number of fluoroscopies, needle placements, and bleeding.

Our study had some limitations. First, most of the included studies were RCSs, and the level of evidence was not as high as that of RCTs. Second, most robot models are made in China, making the conclusions of this study non-generalizable to other robot models. However, despite these limitations, the RCSs included in this meta-analysis all had scores of seven stars or above and remained of high quality.

## Conclusion

Robot-assisted nailing technique helps improve postoperative healing rates in patients with femoral neck fractures, shortens healing times, restores hip function, reduces the number of intraoperative fluoroscopies, guides pin placements, reduces intraoperative bleeding in patients, and increases perioperative safety than the freehand nailing technique.

### Supplementary Information

**Supplementary Material 1.**

**Supplementary Material 2.**

## Data Availability

All data generated or analysed during this study are included in this published article.

## Abbreviations

RCT: Randomised controlled trial
RCS: Retrospective cohort study
RA: Robot-assisted
FH: Freehand
SMD: Standardised mean difference
CI: Confidence interval
