# Supplementary material for: Comparison of rehabilitation outcomes between robot-assisted and freehand screw placement in treatment of femoral neck fractures: a systematic review and meta-analysis
Source: BMC Musculoskelet Disord. 2024 Mar 8;25:208. doi: 10.1186/s12891-024-07325-0 (PMC10921808; doi:10.1186/s12891-024-07325-0)
Supplement: Supplementary file 1 — Supplementary Material 1. [file 12891_2024_7325_MOESM1_ESM.doc]

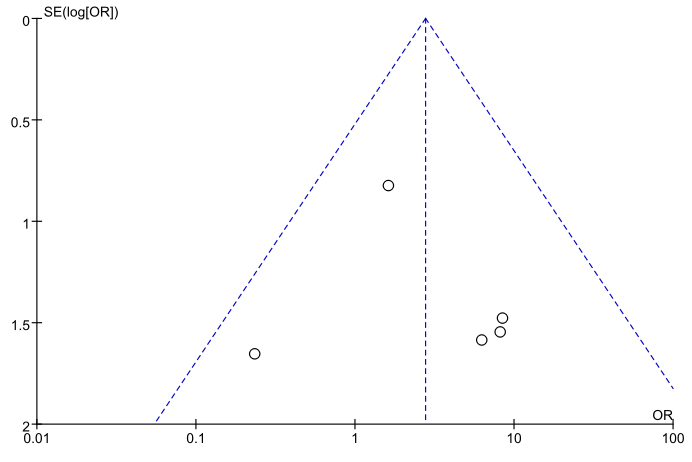


Figure. S1 Funnel plot of healing rate


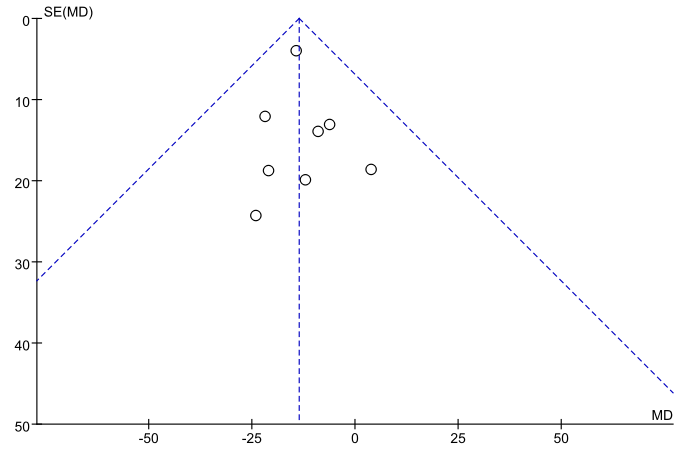


Figure. S2 Funnel plot of length of healing time

Figure. S3
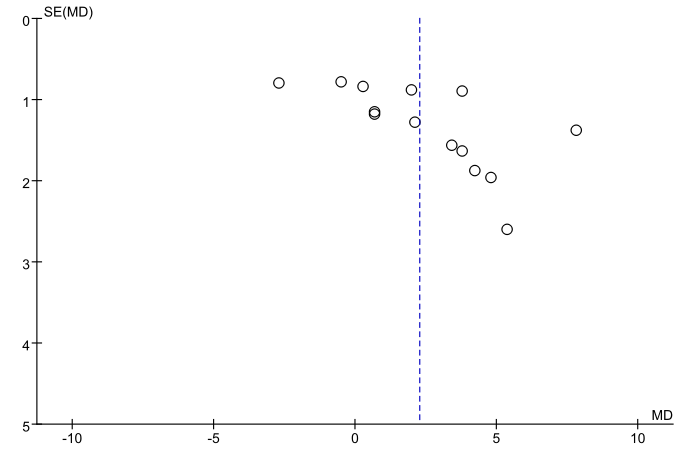
Funnel plot of Harris score


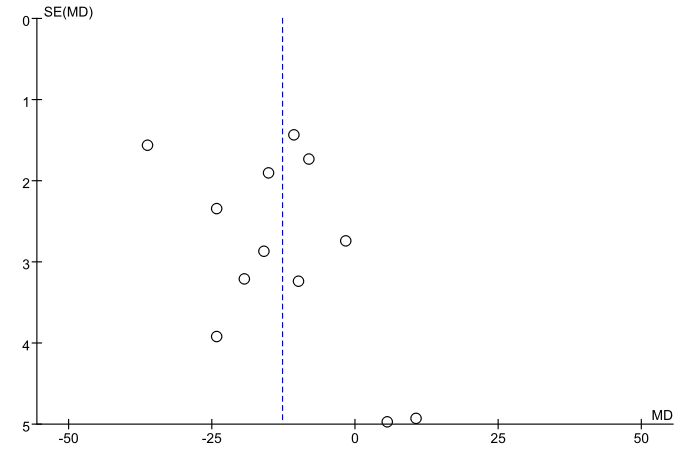


Figure. S4 Funnel plot of operation time


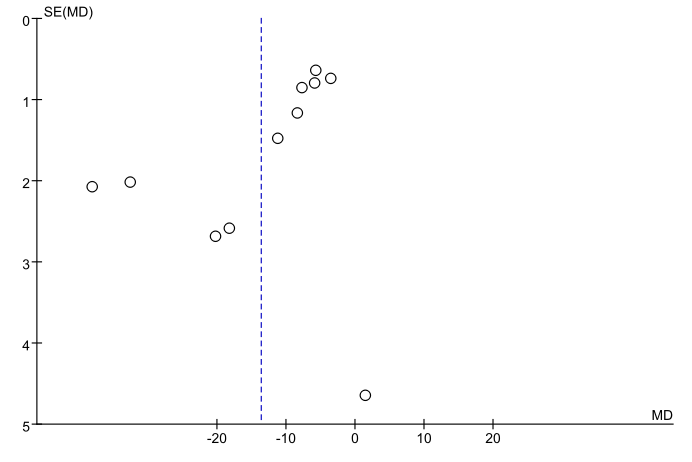


Figure. S5 Funnel plot of frequency of X-ray fluoroscopy


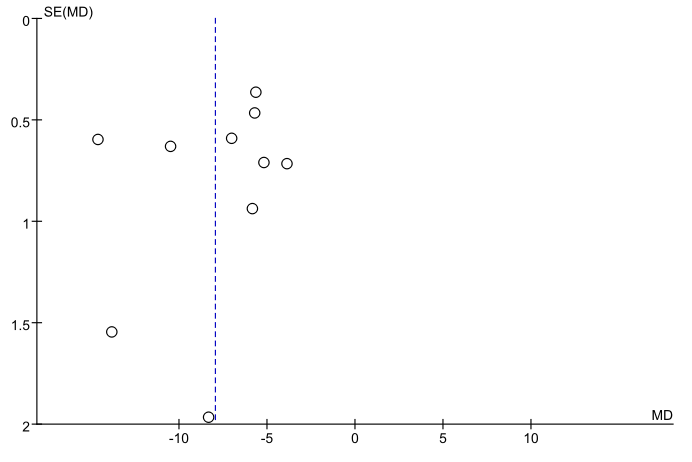


Figure. S6 Funnel plot of frequency of guide pin insertion


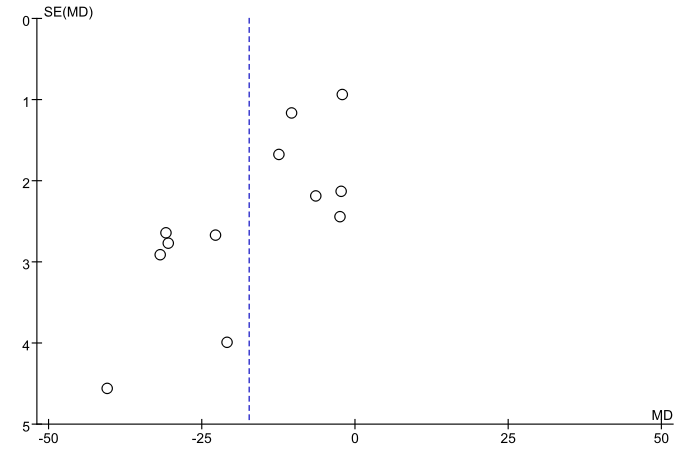


Figure. S7 Funnel plot of Intraoperative blood loss
