# Supplementary material for: Comparison of rehabilitation outcomes between robot-assisted and freehand screw placement in treatment of femoral neck fractures: a systematic review and meta-analysis
Source: BMC Musculoskelet Disord. 2024 Mar 8;25:208. doi: 10.1186/s12891-024-07325-0 (PMC10921808; doi:10.1186/s12891-024-07325-0)
Supplement: Supplementary file 2 — Supplementary Material 2. [file 12891_2024_7325_MOESM2_ESM.pdf]

Editing Certificate

This document certifies that the manuscript listed below has been edited to ensure language and grammar accuracy and is error free in these aspects. The logical presentation of ideas and the structure of the paper were also checked during the editing process. The edit was performed by professional editors at Editage, a brand of Cactus Communications. The author's core research ideas were not altered in any way during the editing process. The quality of the edit has been guaranteed, with the assumption that our suggested changes have been accepted and the text has not been further altered without the knowledge of our editors.

MANUSCRIPT TITLE

Comparison of Rehabilitation Outcomes Between Robot-Assisted and Freehand Screw Placement in Treatment of Femoral Neck Fractures: A Systematic Review and Meta-Analysis

AUTHORS

Li Yiyang

ISSUED ON

January 27, 2024

JOB CODE

ALHWY_4_2

THE EDITAGE Promise QUALITY GUARANTEE

Prabh Grewal
Senior Vice President - Editage

editage helping you get published

Since 2002, Editage has helped over 430,000 authors publish around 1.2 million research papers in scholarly journals across over 1000 disciplines through editorial, translation, transcription, and publication support services. Editage is a brand of Cactus Communications (cactusglobal.com), a science communication and technology company.

CACTUS

GLOBAL :
+1(833) 979-0061 | request@editage.com

CHINA :
400-120-3020或021-6020-9400 | fabiao@editage.cn
